# Free Radical Research in Cancer

**DOI:** 10.3390/antiox9020157

**Published:** 2020-02-15

**Authors:** Ana Čipak Gašparović

**Affiliations:** Division of Molecular Medicine, Ruđer Bošković Institute, HR-10000 Zagreb, Croatia; acipak@irb.hr

It can be challenging to find efficient therapy for cancer due to its biological diversity. One of the factors that contribute to its biological diversity are free radicals. Evolutionary, aerobic organisms evolve in an oxygen atmosphere, improving the energy production system by using oxygen. Oxygen is beneficial, but it can also be detrimental if free radicals are formed [1]. Free radicals, as well as some non-radical species that have oxygen, are reactive oxygen species (ROS). ROS can damage DNA leading to mutations, single, or double-strand breaks [2]. These events, if the cell is unable to repair the damage, are deleterious. If not fatal, these changes in genetic material result in tumor development by losing cell cycle control. Further, these mutations create genetic instability that result in tumor heterogeneity, and thereby increase the possibility of surviving stress conditions. In addition to direct interaction with DNA, proteins, and lipids, ROS are also signaling molecules that take an active part in regulating cellular processes [3,4]. It was previously thought that ROS only damage cells, but we now know that some enzymes primarily produce ROS, and they are not by-products [3]. These are NAD(P)H oxidases (NOX) and they produce ROS in response to inflammatory signaling. This planned production of ROS may play a role in proliferation, as ROS are able to activate signaling pathways, such as mitogen activated-protein kinase (MAPK)/extracellular-regulated kinase 1/2 (ERK1/2), phosphoinositide-3-kinase (PI3K)/protein kinase (Akt), and more, thoroughly reviewed in [3]. An important factor in surviving ROS is the antioxidant system of the cell. The main role of this complex system is to remove the excess ROS. As there are many ROS, there are many different parts of this system acting in a similar or unique way in removing ROS, such as the glutathione system, superoxide dismutase-catalase catalase, thioredoxin system, and small molecules (e.g. vitamin C, vitamin E). In order to ensure the right levels of these enzymes and small molecules, ROS activate several antioxidative transcription factors, such as Nrf2 and the FoxO family. These transcription factors are responsible for activating the majority of antioxidative genes [5,6,7]. Generally, cancer cells have increased amounts of ROS; consequently, they adapt by increasing the antioxidative defense system [8], thereby, strongly linking ROS and antioxidative research.

Nevertheless, ROS were at first considered detrimental, and this was used as a therapeutic strategy in fighting cancer. Most of the conventional types of chemotherapy, as well as radiotherapy, are based on ROS production. Unfortunately, this strategy has to eradicate the tumor completely, otherwise the surviving cells adapt and build up their antioxidant systems, as well as other mechanisms (e.g., drug transporters) making themselves resistant. Strategies involving activation/inhibition of signaling pathways (and here, Nrf2 was certainly an attractive target) turned out to be a double-edged sword [9].

This Special Issue aims to provide different approaches to study the role of free radicals in cancer. Recent findings are presented within eight original papers and four review papers, spanning from cancer therapy and resistance development to side effects of cancer therapy, with its effects on human health, in a process governed by free radicals.

The focus of the review papers is on free radicals, ROS, and cancer therapy. As mentioned above, ROS modulate cellular signaling pathways and are therefore important to maintain redox homeostasis. A review by Kim et al. [10] provides an overview of cellular ROS production, both controlled and uncontrolled, as well as ROS elimination (keeping in mind the importance of this homeostasis). Further, redox changes in cancer are described, with emphasis on chemotherapy based on ROS production. The paradox of chemotherapy is discussed: the chemotherapy resistance can be acquired through either increased proliferation (leading to resistance) or by changing to a cancer stem-like cell phenotype, with a low proliferation rate. In hand with this review is the work of Mendes and Serpa [11], which discusses metabolic remodeling of lung cancer. These metabolic changes occur via several mechanisms, which include mutations, as well as responses to oxidative or alkylating treatments. These events lead to chemotherapy resistance that occur because of changes in drug transporters, as well as in antioxidants. Metabolic remodeling is therefore a challenge in cancer therapy, and can be used—if the changes are well monitored and defined—to adapt to clinical therapy, in order to avoid recurrence.

The review papers by Clavo et al. [12], and Prasad and Srivastava [13], discuss adjuvant cancer therapy by reduction of ROS. Natural compounds, such as Triphala and Ayurvedic medicine, have antioxidative properties, and prevent free radical formation and lipid peroxidation. In addition to antioxidative properties, the authors also discuss the chemopreventive and chemotherapeutic effects of Triphala, which are encouraged by the results of three clinical studies. Another strategy in fighting cancer, described by Clavo et al. [12], is the use of ozone as an adjuvant therapy to conventional chemotherapy. The authors present evidence of beneficial effects of ozone therapy on animal models and describe possible mechanisms by which these effect may occur.

Mechanisms, by which cellular processes are changed in cancer, spread on different molecules (such as enzymes, transcription factors, or ion channels). An example of an ion channel is the transient receptor potential melastatin 2 (TRPM2), a Ca^2+^ channel that can be activated by H_2_O_2_ [14]. A study presented by Hack et al. [14] showed parallel expression of NOX4 and TRPM2 in human granulosa cell tumor samples, suggesting that induction of oxidative stress could be beneficial for the therapy, as activation of this channel by H_2_O_2_ increased Ca^2+^ levels and apoptotic cell death.

Acquired resistance was a model in two papers and was achieved through growth of cells under conditions of chronic oxidative stress. Both models used breast cancer cell lines in their study. In a study by Glorieux and Calderon [15], NQO1 affected cancer redox homeostasis and sensitivity to drugs. Consequently, NQO1 polymorphism may be used as an important factor if quinone-based chemotherapeutic drugs are considered as cancer therapy. Interestingly, NQO1 is a target gene for NRF2, an antioxidative transcription factor. Using breast cancer cell lines stimulated for cancer-stem-like phenotypes under chronic oxidative stress, we showed an increase in NRF2, but also in some epithelial-mesenchymal transition markers, indicating that NRF2 can play a role in breast cancer resistance [16]. 

In addition to breast cancer, ROS and NRF2 were studied in regards to the androgen receptor and its splice-variant AR-V7 [17]. As therapy for prostate cancer, a triterpenoid antioxidant drug was tested for its ability to regulate androgen receptor expression. This drug proved to enhance efficacy of clinically approved anti-androgen, but also decreased ROS and increased NRF2, indicating possible mechanisms of action. There are numerous consequences of prostate cancer therapy due to ROS production, but effects on sperm are not fully investigated. Takeshima et al. [18] show evidence that cancer chemotherapy has similar effects on semen as idiopathic infertility, suggesting antioxidant therapy to reduce ROS.

As mentioned, many conventional cancer therapies are based on free radical/ROS production. Photodynamic therapy is also a cancer therapy that uses chemosensitizers to generate free radicals, which then act against the tumor. Such a photosensitizer, a tailored boron-dipyrromethene (BODIPY) derivative, was used on A375 and SKMEL28 cancer cell lines [19]. Authors show positive effects of this compound by inducing singlet oxygen and NO to cause cell death.

Finally, Rodríguez-García et al. [20] studied protein carbonylation in patients with myelodysplastic syndromes. These patients had increased protein carbonyls, but levels decreased after treatment with an iron chelator (deferasirox). Analysis of the p21 gene expression in bone marrow cells revealed correlation between high protein carbonyls and increased expression, and vice versa. The paper suggests that the fine-tuning of oxidative stress levels in bone marrow can determine the disease progression in these patients.
